# Population and Health Policy Considerations

**DOI:** 10.1093/geroni/igaa057.3149

**Published:** 2020-12-16

**Authors:** Julie Robison

**Affiliations:** University of Connecticut, Farmington, Connecticut, United States

## Abstract

The risk of death, disease, disability, hospitalization, institutionalization and high health care costs varies among individuals with increasing heterogeneity associated with aging. Frailty, physical performance measures, self-reported measures and multimorbidity all represent measures that are useful in helping to better define such heterogeneity at the level of populations and to ultimately define such risk in individuals. These higher risk individuals account for a growing proportion of this nation’s health care costs, with continued increases over time that appear unsustainable in the long term. Therefore, efforts to better define the nature of such heterogeneity of risk and improved targeting, with the goals of improving outcomes and reducing costs, are essential. A closely related challenge is to effectively translate proven clinical and health system interventions from the world of research to that of health policy and real-world clinical practice via pragmatic trials.

